# Establishing the Durability and Reliability of a Dental Bur Based on the Wear

**DOI:** 10.3390/ma16134660

**Published:** 2023-06-28

**Authors:** Filip Ilie, Ioan Alexandru Saracin

**Affiliations:** 1 Department of Machine Elements and Tribology, University Politehnica of Bucharest, 060042 Bucharest, Romania; 2 Department of Motor Vehicles, Transport and Industrial Engineering, Craiova University, 200585 Craiova, Romania; saracinalex@gmail.com

**Keywords:** dental bur, dental material, durability, polynomial interpolation, reliability, wear

## Abstract

This paper analyzes the phenomenon that conditions the durability and reliability of a type of dental bur based on the wear of the active part and with effect on its quality. For the experimental study, a conical-cylindrical dental bur and a sample dental material in cobalt–chromium alloy, cylindrical shape, tested on a specially made experimental installation were used. In this paper, the most significant parameter was considered (loss of mass, *m_w_*, through the wear of the active part of a tested dental bur), which highlights the studied wear phenomenon. This is useful for the establishment of the durability and reliability of the dental bur by the extension of the lifetime or even optimization of its operation. The wear phenomenon of the active part of dental bur is studied based on the results and experimental data obtained in the work process that was validated by interpolation and led to polynomial functions which approximate very well the dependent parameter, *m_w_*, considered in the experimental program. The results of the interpolation showed that in the first 11 h of work, the dental bur works with high efficiency (allow optimizing operation or offering new ideas for constructive solutions), after which it can be easily decommissioned; i.e., it should be replaced with a new one (establishing some possible criteria for replacing the used dental bur).

## 1. Introduction

Durability and reliability for a technical system represent the main conditions for an effective and defect-free operation. According to [1], durability means the period of time that a technical system can be used in operation under previously established conditions, i.e., the quality of the materials to maintain their initial properties after the period of operation.

Reliability is a quality characteristic, or the ability (safety in function/exploitation) of use over time of a technical system, that is, not to break down for a set period of time under working conditions and in compliance with prescribed norms [1,2].

As a result, reliability has to be measured, forecasted, and ensured through programs aimed at increasing product quality. The most modern description of technical systems, which lends itself very well to the calculation of reliability, is the systemic one. 

Mathematically, this represents the essential component of the safety in operation/exploitation of the system (without failures, of all elements), under known working conditions that actually represent the reliability, maintenance, availability, and safety of the system.

As a time function, reliability is defined as the probability of failure-free operation in which the pre-set parameters in time range [0, t) between the values of 0 and 1.

In the case of complex systems, the reliability of each component element is considered for the calculation of operational safety, and in the case of technological processes, it refers to ensuring the volume of production while maintaining the quality of the products over time and in correlation with the established requirements.

Practically, by calculation, the predictive reliability is determined (projected, established during the design of the product [3]), which is verified in the laboratory, that is, the experimental reliability is obtained; in operation, the operational (effective) reliability is confirmed; the product, by design theme, can be written and possibly, the nominal reliability; and with the start of the product’s operation, the potential reliability is established (also experimental) on the elements resulting from series production [4] according to ref. [5]. So, the reliability activity becomes an important component of complete quality control (that is, quality control passes through all forms of reliability), respectively, of the process of designing, experimenting, manufacturing, and operating a product or system.

The research in this work refers only to the active part of the dental bur. If one were to consider the whole dental bur, other component elements should also be considered: the drive motor, the bearings, the clamping systems of the dental bur, etc. The defects of the active part of a dental bur must be studied experimentally, together with its wear [6,7].

The active part of the dental burs is the component without automatic restoration (non-repairable), [5], after the failure or loss of working capacity, i.e., once worn, a dental bur is no longer repaired, but replaced/changed.

The defect is a deviation from quality which appears during operation, and for the case under investigation, it represents the inability of the active part of the dental bur to operate at the parameters required in the work process/experiments and imposed by the designer, or to perform the ordered functions, respectively, without interruption of operation in the interval of exploitation time.

According to [5], defects are the starting points of some disturbances, inconveniences, material, physical, or moral imperfections, etc., and may appear during the execution phase being caused by the material, the equipment used, the user, etc., and during exploitation they are caused by deformations, shocks, the working environment, wear, etc. Quantitative failures can be determined by adding up the times of good operation of the system [3].

So, with dental burs being fine mechanical tools used for technically delicate operations and being made of expensive materials, rapid wear has led to studies and research in the hope that an extension of their life or even an optimization of their operation can be achieved. Therefore, in this paper study and research were undertaken for practical applications with the aim of extending the life of dental burs, optimizing their operation, and establishing possible criteria for replacing used dental burs. This has been achieved through a detailed analysis of the phenomena that limit the durability and reliability of dental burs, both analytically and experimentally, based on the wear behavior of the active part of dental burs in the process of operation. The wear behavior of the active part of a dental bur is studied based on the results and experimental data obtained in the work process and presented mathematically in the form of polynomial interpolation functions, of at most the third degree. The polynomial interpolation functions are only valid in the testing conditions of the dental bur to describe the wear phenomenon, and the extrapolations do not lead to satisfactory results. At the same time, they lead to practical applications, such as extending the life of the dental burs (including the materials from which the dental burs are made), which is useful even for optimizing their operation, determining possible criteria for replacement of used ones, or providing ideas for constructive solutions.

In this paper, to evaluate the wear in the work process of the active part of the analyzed dental bur, the deterministic differential model was used. 

However, none of the critically reviewed studies used the rotational velocity–time–wear (mass lost due to wear) characteristic, as in the present paper. This model allows the extension of the characteristic on other parameters of the dental bur that change in the working process as a result of wear. In addition, the methodology used is a novelty in the field, although in the literature there are various models of experimental data, considering one, two, or even three parameters, in other forms and applying other mathematical methods. 

By the study of durability and reliability based on the wear phenomenon for the dental burs (of the same type) tested and by the experimental data, it has been shown that practical results can be obtained, such as the extension of the lifetime of the dental bur (and of the material from which is built) by one hour (compared to 10 h of the literature, i.e., with a percentage increase of 10%) or even working process optimization of the dental bur in operation. 

The experimental results obtained for the wear of the active part of dental burs in the work process were validated by interpolation and led to polynomial functions which estimate very well the considered parameter, *m_w_*. The interpolation results established the time range in which the dental bur works with high efficiency, after which it can be easily decommissioned, i.e., it should be replaced with a new one. In another order, the experimental results for the parameter, *m_w_*, measured by weighing, took different values (randomly) between two limits with an own frequency, and their distribution is according to Gauss’s normal law. At the same time, the results allow the optimization of the operation, improvement of other characteristic/functional parameters, and determination of some possible criteria for replacing the used dental bur or offering new ideas for constructive solutions.

Therefore, the aim of the paper is to study the phenomenon of wear by establishing the durability and reliability of the active part of a dental bur, as an important issue regarding the use, productivity, and financing of dental services.

## 2. Materials and Methods

**Figure 1:**
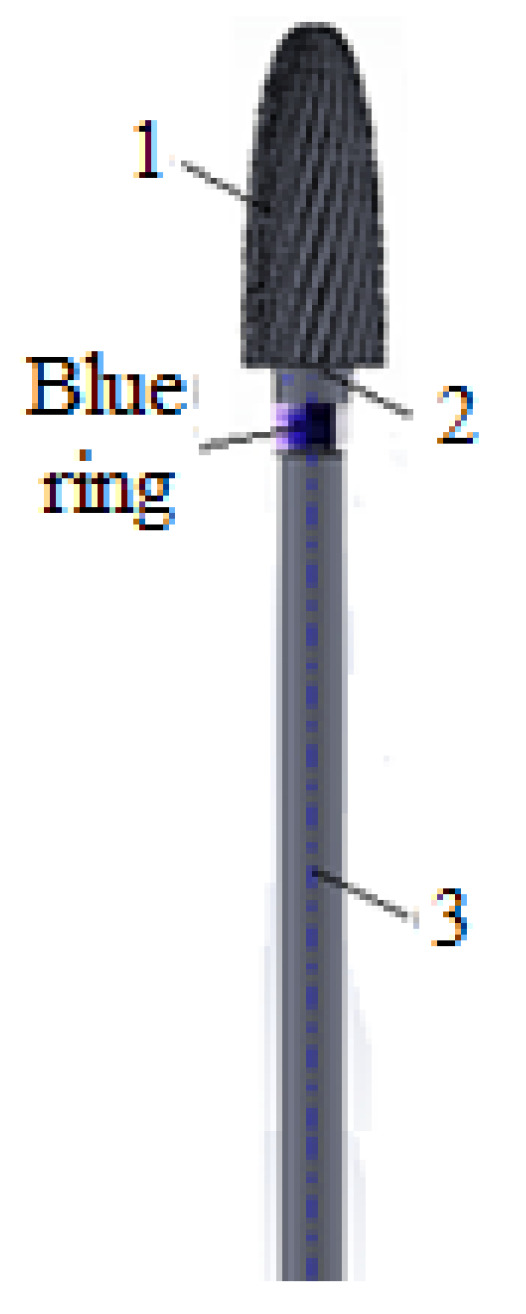
Conical-cylindrical dental bur: 1—the active part of the dental bur, 2—the neck of the dental bur, 3—dental bur foot.

For research and experimental determinations, a conical-cylindrical dental bur [8] was used (of type DFS Diadur Quattro 603501 from the company DFS-Diamon GmbH, Riedenburg, Germany) and is shown in Figure 1. In the experimental tests, 20 dental burs were used of this type, distributed in four groups of five, with each group being tested at a different rotation speed. The dental bur is made of a metallic mixture in which tungsten carbide (WC) dominates together with other carbides of Cr, Mn, Fe, etc. with the medium graining on the active side/part and it is used for processing Co-Cr alloys, as well as other semi-precious and precious metals; it has a blue ring for identification and works at speeds up to 35,000 rpm [9]. This type of dental bur is considered a sample/specimen with the following sizes: total length of 52.75 mm, length of the active part of 14.50 mm, maximum diameter of 6.20 mm, average diameter of 5.40 mm, and a mass of 4.701 g. 

**Figure 2:**
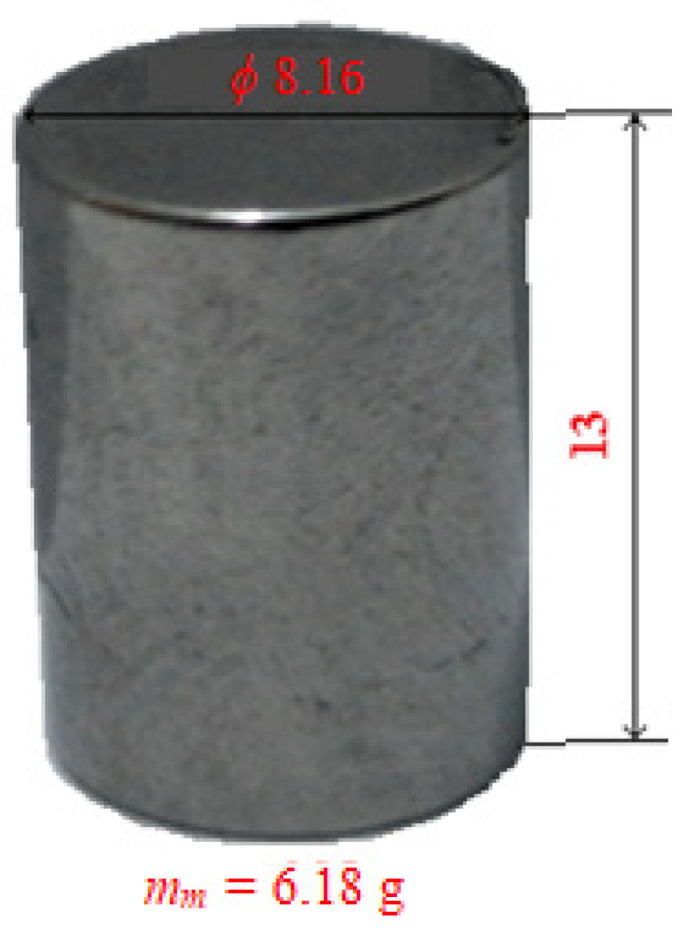
Sample dental material (in Co-Cr alloy).

The sample dental material used for the experimental tests was made of cobalt–chromium alloy (Co-Cr) because it is a material for dental work in dentistry and is presented in Figure 2, having a cylindrical shape with a diameter of 8.16 mm, a length of 13 mm, and a mass of 6.18 g.

**Figure 3:**
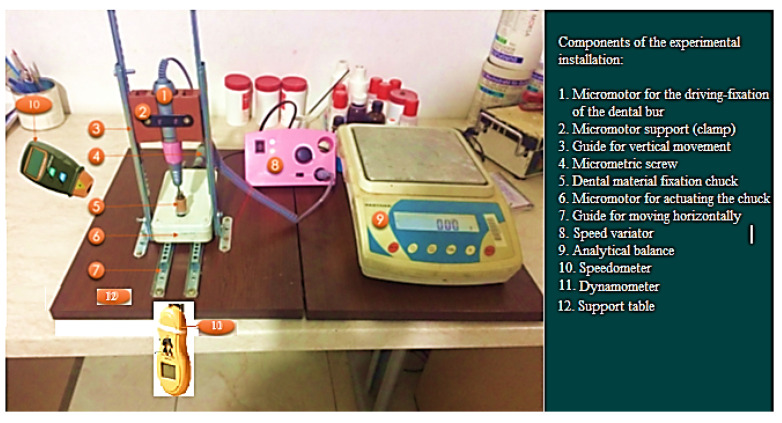
The experimental installation with the component elements [10].

In addition, to perform the experimental tests, the experimental installation shown in Figure 3 was created and used. The operation of the experimental installation is considered simple and consists of a micromotor for fixing and driving the dental bur (1), the support micromotor (2) for fixing the dental bur assembly with the clamp, the vertical movement of the micromotor (1) on the vertical guide (3), the micrometric screw (4) that measures the movement of the chuck that fixes the material to be milled (5) and is driven by the micromotor (6), which moves horizontally on the also-horizontal guide (7). Establishing the speed of the dental bur for milling the dental material and its variation is obtained with the help of the speed variator (8). The specimen/samples (dental burs and dental material) are measured and weighed both before and after testing with an analytical balance (9). A tachometer (10) and dynamometer (11) were used to measure and read the speed rotation of the dental bur and the material to be milled, respectively, as well as the pressing force between the dental bur and the material to be milled; and the test time is measured with a stopwatch. All the components of the experimental installation are fixed and placed on a support table (12).

To start the experimental tests, preliminary preparation of the experimental installation was necessary, by selecting the type of dental burs (see Figure 1) and the dental materials used in the experimental determinations (see Figure 2). These were previously weighed, and the parameters of the work process were defined, with notations, units of measurement, and their type, according to Table 1.

After establishing the working regimes (rotation speed, time, pressing force, advance speed) for the experimental research, the dental burs were fixed in a row in the micromotor support chuck (1) (see Figure 3), which ensures the rotation movement at the preset working regime, and the dental material in the support chuck (5) of the micromotor (6) (see Figure 3). Then, the pressing force between the active part of the dental bur and the dental material (of 40 N), the rotational speed at which the milling is performed (of 7000, 12,000, 20,000, and 35,000 rpm), and the test time (of 1, 2, 3, 3.5, and 4 h) were fixed.

These were followed by setting the rotation speed of the dental material to be milled and its advance speed (of 1 mm) by operating the micrometric screw (see Figure 3). Then, the experimental installation (see Figure 3) was put in the operation and five tests were performed in each group and for each of the established working times and the rotational speeds. It is mentioned that the experimental results/data were stored in an Excel numerical database. At the end of the time initially set for the testing for each of the established speeds, the dental burs used for the experimental tests were weighed again.

For the experimental tests, the 20 dental burs (of the type in Figure 1) were used, distributed in groups of five for each of the four rotation speed regimes. This number (of 20 dental burs) is considered to be a sufficient minimum for the experimental program and to draw conclusions about the wear evolution, useful for establishing the durability and reliability of the researched dental bur. The pressing force (of 40 N, representing the maximum force developed (achieved) by the experimental setup in Figure 3, specially designed and made for the experiments) and feed rate (1 mm) were kept constant.

The experimental method used, regarding the wear behavior of the dental bur chosen as a specimen (conical-cylindrical), considered both the properties of the dental bur material and those of the processed dental material, as well as the parameters of the work process (rotation speed, pressing force, feed, and test time).

The data/result analysis method is based on: the polynomial interpolation of the experimental results of the parameter, *m_w_*, to describe the wear phenomenon of the active part of the dental burs and for the evaluation of wear and lifetime (durability), using the least squares technique; the estimate the durability or lifetime of the active part of dental bur by the working time calculation; determining by calculation of the reliability of the dental bur considering the reliability–durability correlation, respectively, the correlation between analytically results and experimental ones, to confirm the veracity of the experimental results.

## 3. Results 

### 3.1. Estimation of Wear and Durability of Dental Burs

The experimental research had in mind, first of all, the determination of the mass, *m_f_* of the specimen dental bur subjected to the research, respectively, of the mass of the processed dental material, *m_m_*, before testing.

Table 2 and Table 3 show the mass and dimensions of the specimen dental bur (Table 2), respectively, and the mass and dimensions of the sample dental material subjected to testing (Table 3), before testing.

The results of the experiments show the dependence on time and the rotation speed of the dental bur, the geometric characteristics of its active part, and the mass of material lost through wear, *m_w_* [10]. The list of these parameters is given in Table 1.

The method used to obtain the results is based on polynomial interpolation using the least squares technique. Thus, the general mathematical relationship of interpolation polynomials, written in canonical form, is as follows:

(1) $$q\left(t,\omega \right)=\sum_{i=0}^{3}\sum_{j=0}^{3}c_{ij}t^{i}\omega^{j},$$

which, developed up to the third degree, becomes:

*q*(*t*, *ω*) = *c*_00_*t*^0^*ω*^0^ + *c*_10_*t*^1^*ω*^0^ + *c*_20_*t*^2^*ω*^0^+ *c*_30_*t*^3^*ω*^0^+ *c*_01_*t*^0^*ω*^1^+ *c*_02_*t*^0^*ω*^2^ + *c*_03_*t*^0^*ω*^3^, (2)

as being sufficient to describe the wear phenomenon of the active part of the dental burs, and *q* the interpolated parameter (here, the mass lost by the wear of the active part of the dental bur, *m_w_*). 

*Note:* As interpolated parameters any other characteristic/functional parameter of the active part of the researched dental bur can be used, for example, seating angle, clearance angle, sharpening angle, the area, radius of the tip circle, etc.

The synthesis of the results of the least squares interpolation for the measured parameter (see Table 1) during the experimental tests is given in Table 4 and they contain the coefficients of the polynomials, *c*_00_*…c*_03_ with two variables (time, t, and the rotation speed of the dental bur, ω, implicitly and of its active part), as well as the average of the squared errors, *ε*, which ranks the approximation performances.

It can be seen that the polynomial coefficients are double indexed, the first index (example: 1 from *c*_10_) represents the power exponent of the temporal variable (time, *t*). The second index (example: 0 of *c*_10_) represents the rotation speed, *ω*.

To calculate the error, *ε*, the relation was used:

(3) $$\varepsilon =100\cdot \frac{\sqrt{{\left(y_{w}-y\left(x_{w}\right)\right)}^{2}}}{\overset{-}{y}\cdot N}$$

where: *y_w_,w* = 1,…, *N* are the experimental data for the dependent variable, for example, *m_w_*; *x_w_* = *t_w_,ω_w_* is the data string of the independent variable, for example, *t* and *ω*; *N* is the number of experimental data; $\overset{-}{y}$ is the mean value of the experimental data for the string of the dependent variable.

For cutting, chipping, etc., of instruments/tools in general and dental burs in particular, the assessment of wear and lifetime (durability) is done in relation to one or more geometric or physical parameters (such as the lamellae angles of the active part of the dental burs, the area, or radius of the peak circle), as described in the literature of the specialty [11,12]. 

**Figure 4:**
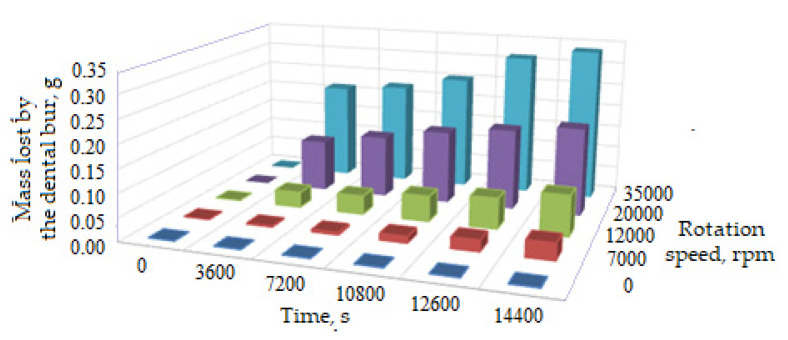
Evolution of the mass, *m_w_* of the active part of dental burs removed by wear in the work process, as a function of time, *t* and of rotation speed, *n*.

Thus, the main indicator of dental bur wear is the mass removed (lost) through wear, *m_w_*. For the experiments carried out, the variation of this parameter, *m_w_*, was recorded for the five operating times, established in advance (of 1, 2, 3, 3.5, and 4 h of operation) and for the four values of the speed rotation of the dental bur (of 7000, 12,000, 20,000, and 35,000 rpm). The results were stored in the Excel numerical database and a synthetic representation of them can be seen in Figure 4.

Similar results can be obtained for other characteristic/functional parameters of the active part of the researched dental bur (the lamellae angles: of seating, clearance, sharpening; the area or the radius of the peak circle, etc.).

Estimation of the lifetime (durability) of the active part of the tested dental bur, cylindrical-conical type (see Figure 1), manufactured from a metal mixture in which tungsten predominates (58.14%), in relation to the mass lost through wear of the active part of the dental bur, *m_w_*, is shown in Table 5. To estimate the lifetime of the dental bur in relation to *m_w_* at the four revolutions used (see Table 5), the experimental tests considered the polynomial interpolation functions of the first, second, and third degree, as specified above.

Thus, to estimate the durability or lifetime of the active part of a dental bur by calculation, the working time calculation relationship was used [13]:

(4) $$t_{\mu }=\frac{\ln\frac{1}{1-\mu }}{\omega \cdot d}$$

established based on the conventional determination of a fraction, *μ* of the total mass of the active part of the dental bur, which is lost during the working process, *ω* the angular velocity of the researched burs, and *d* model parameter, which is determined by the method of least squares applied to a non-linear function dependent on *m_w_* and the angular space (friction length) traveled by the dental bur.

**Figure 5:**
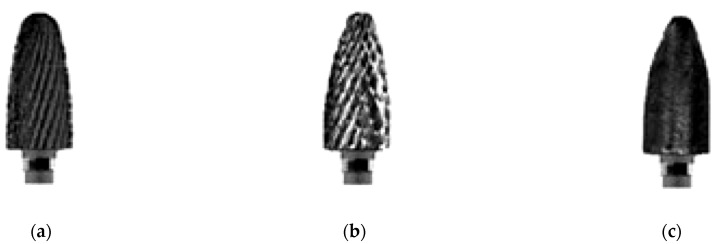
Determination of the mass of the circular crown of the active part of the dental bur: (**a**) specimen dental bur, after 1 h of operation; (**b**) worn dental bur after 4 h of operation; (**c**) dental bur with the circular crown of the active zone, completely worn.

To establish the critical fraction, *μ* of material of the active part of the dental bur, which can be lost by the milling process, one can start from about 65% of the mass of the approximate circular crown of the active part of the dental bur, determined experimentally through the total wear of the lamellae of the active part of the dental bur, followed by weighing; by the difference to the weight of the specimen dental bur (Figure 5), the value of 0.522 g was obtained.

Thus, for the dental bur tested in the laboratory, the results are those given in Table 6 from the column of mass lost through wear, *m_w_*.

**Figure 6:**
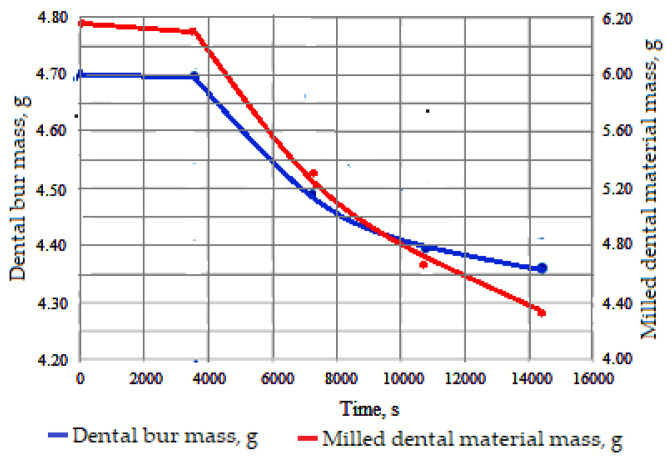
Time variation of the dental bur mass lost and the dental material in the working process.

Using the experimental data whose evolution can be seen in Figure 6, the values obtained are for the time variation of the mass of the dental bur, *m_w_*, and of the milled dental material, *m_m_*. Thus, the initial mass of the specimen dental bur was *m_f_* = 4.701 g, and the mass of the processed dental material was *m_m_* = 6.180 g; the final values of the mass of the dental bur, *m_w_*, varied between 4.700 g and 4.360 g and of the processed dental material varied between 6.102 g and 4.160 g. Figure 6 shows the average variations in time of the masses of the two bodies in interaction/contact with relative movement (in the work process): the dental bur and the dental material, subject to milling. The processes that take place by friction on the contact surfaces and in the surface layers, respectively, in the micro volumes of asperities in contact are of a different nature and depend on a series of mechanical, physical, chemical, and geometric factors.

It is observed that both bodies (dental bur and dental material), which interact (are in contact with relative movement), lose mass. The loss of mass over time has a variation according to an exponential curve (after one hour of operation), even if, in the prescribed working interval, due to the values of the parameters, their curvature is not sufficiently accentuated.

For the mass lost by the dental bur and the mass lost by the milled dental material, the efficiency ratio of the dental bur was additionally considered, *e*(*t*), i.e., the ratio between the dental material lost (removed, in grams) to the material lost (removed, in grams) by the dental bur.

**Figure 7:**
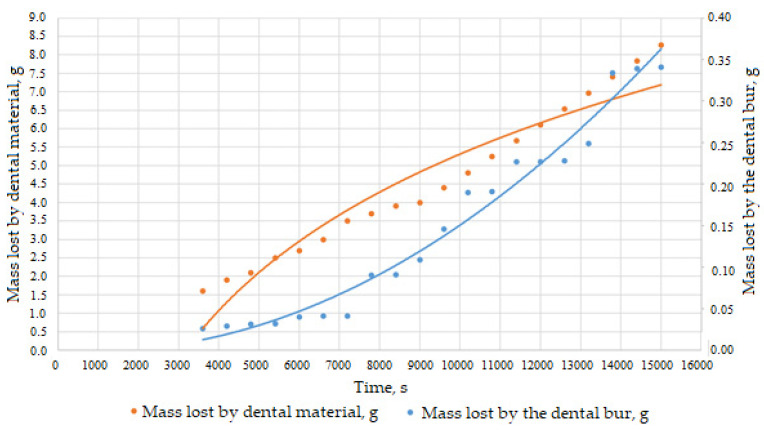
Time variation of mass losses of milled dental material and dental bur, measured experimentally.

**Figure 8:**
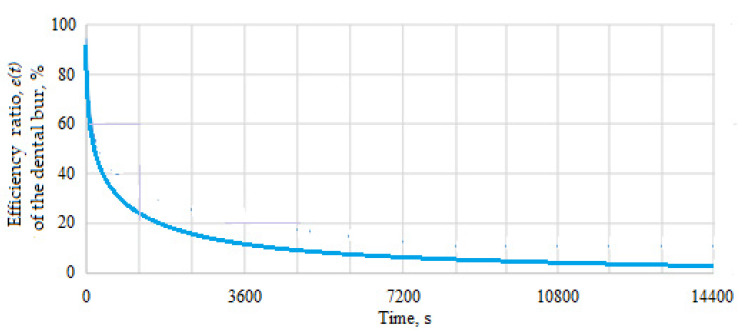
Time variation of the efficiency ratio *e*(*t*) between the mass loss of the milled dental material and the mass loss of the dental bur material.

The graph of the variation of the mass of material lost by the dental bur and of the milled dental material as a function of time, measured during the tests, can be seen in Figure 7, and the time variation of the efficiency ratio *e*(*t*) between the mass loss of the dental material milled and the mass loss of the dental bur material (on the active area) is represented in Figure 8.

Based on the graphical representations in Figure 7 and Figure 8, it can be seen that the material and configuration of the dental bur allow it to be efficient, meaning that a small amount of dental bur material lost removes more material from the milled dental material. However, this effect is visible for a time much longer than four hours of operation, after which the inefficiency of the dental burs was observed (see Figure 5).

### 3.2. Reliability of Dental Burs: The Reliability–Durability Correlation

Considering the experimental results, and in addition those obtained from practice from users of dental burs, who use/consume a lot of dental burs, it turned out that:

- The active part of dental burs operates with a very low probability of failure until a time, *t_min_*, after which it increases rapidly until a time, *t_max_*, when practically no bur is usable anymore, in general;
- The time interval for removing from use the active part of a dental bur, *t_max_-t_min_*, is due to the small differences between the manufacturing characteristics of the same type of active part of dental burs (slightly different masses, angles, etc.).

These differences ultimately have implications for lifespan, as seen in the durability calculation. The interval of increasing failure probability [*t_min_*, *t_max_*] is greater when the differences between the active parts of the same type, even from the same set of products are larger. With these two observations and using the definitions of the “cap” and “bump” type functions, a reliability function *R*(*t*) (probability of correct operation at a time *t* ≤ *t_max_*) is proposed for the operation of dental burs, having the expression [14]:

(5) $$\begin{array}{l} R(t)=\left\{\begin{array}{l} \exp\left(\frac{1}{t_{\max}^{2z}}\right)\cdot \exp\left(\frac{1}{t^{2z}-t_{\max}^{2z}}\right),\;0\leq t\leq t_{\max}\\ 0,\;\;t>t_{\max} \end{array}\right., \end{array}$$

where *t_max_* is the time after which it is experimentally established that the active parts of the dental burs of the same brand and the same type have failed, and *z* is a parametric exponent that can be chosen, so that the time, *t_min_*, at which starts the exponential increase in the probability of failure, is to be as best estimated as possible. Corresponding to the reliability function, *R*(*t*), the failure function, *F*(*t*), and the frequency function, *f*(*t*), are also defined, as useful for interpreting the results.

In this case, a satisfactory estimate is obtained for *z* = 0.75, the value for which it is established as realistically as possible the minimum time (*t_min_* = 10,265 h), from which the failure begins (the exponential increase of the failure probability) of the active part of the dental bur. Under these conditions, for the tested dental burs whose characteristics are given in Table 2, at their speed of 7000 rpm (for example), the times *t_min_* = 10,265 h, respectively, *t_max_* = 12,613 h were estimated according to Table 6, from experimental determinations.

**Figure 9:**
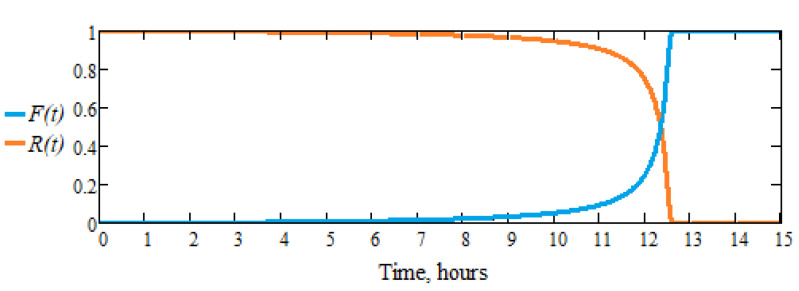
Graph of the failure function, *F*(*t*), and the reliability function, *R*(*t*), for the active parts of the tested dental burs.

**Figure 10:**
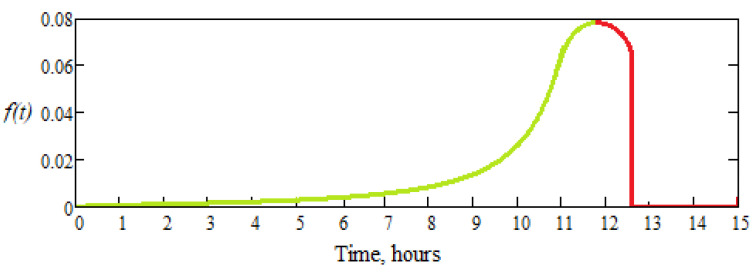
Graph of the frequency function, *f*(*t*), for the tested dental burs. 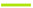 the probability that the dental bur, in good working condition until the moment when the failure begins. 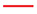 the probability density of failure of the dental bur and the moment in which it is fails.

In Figure 9 and Figure 10, the failure function graph, *F*(*t*) (distribution or probability of failure and *F*(*t*) = 1 − *R*(*t*), *t* ≥ 0) and frequency, *f*(*t*) (density of failure probability, f(*t*) = *F’*(*t*) = *dF*(*t*)/*dt* = − *dR*(*t*)/*dt*, (*t* ≥ 0), corresponds to the reliability function, *R*(*t*), from the relation (5).

It is observed that the reliability function *R*(*t*) is monotonically decreasing, with values in the interval [0, 1] and when time tends to infinity (*t* → ∞), *R*(*t*) cancels (even for finite values, i.e., *t_max_*, in our case, *R*(*0*)) = 1 and when *t* → ∞), *R*(*t*) = 0). The failure function *F*(*t*) is increasing, with values in the interval [0, 1], *F*(*0*) = 0, and when *t* → ∞), *F*(*t*) = 1, and the frequency function, *f*(*t*); check the relationship $\int_{0}^{\infty }f\left(t\right)\;dt=1$.

**Figure 11:**
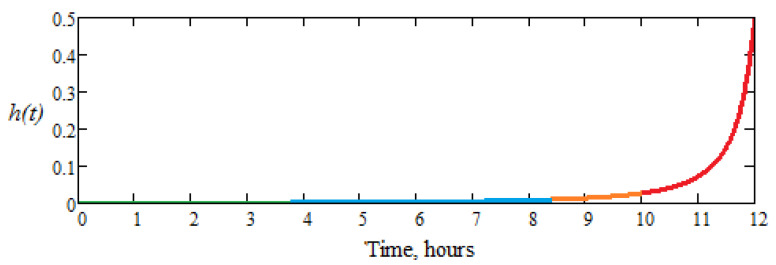
Graph of the instantaneous risk of failure function, *h*(*t*), for the group of investigated dental burs. 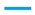 the area where there is no instantaneous risk of failure. 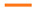 the area where the phenomenon of wear becomes visible, and the risk of failure increases curvilinear (after a curve). 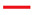 the area where the risk of failure increases suddenly (exponentially and the risk of failure is very high).

Figure 11 shows the function *h*(*t*) (which measures the instantaneous risk of failure and *h*(*t*) = *f*(*t*)*/R*(*t*), *R*(*t*) ≠ 0) and it is observed that for 11 h, the risk failure rate is below 10%. In the 12th hour of operation, the risk of failure (which in the case of the active part of the dental bur means putting it out of action) increases approximately exponentially, that is, after 11 h of operation, which means that it is normal to replace the dental bur.

In addition, the function *h*(*t*) has an evolution that we can divide into four areas:

- The first zone, when *t* Є (0, 4) hours; there is no risk of failure, and *h*(*t*) has a linear variation, close to zero;
- The second zone, when *t* Є (4, 8) hours; there are minimal (very low) chances of failure, although *h*(*t*) has a linear variation with a very small slope;
- The third zone, when *t* Є (8, 10) hours; *h*(*t*) begins to increase, has a curvilinear variation, so the phenomenon of wear becomes visible, and the risk of failure is increasing;
- The fourth zone, when *t* Є (10, 12) hours; *h*(*t*), increases suddenly, following an exponential curve and the risk of failure is very high.

So, the researched dental burs can work without major risk of failure for up to 10 h, which is very close to those calculated analytically based on the experimental results, that is, this confirms the veracity of the experimental results and the correlation between analytically results and experimental ones.

## 4. Discussions

Due to the small relatively number of results, no superior method of analysis can be used; nor through the method approached (the polynomial interpolation using the least squares technique) can interpolation polynomials higher than degree III be obtained. The polynomial interpolation functions allowed the determination of some characteristic points, which represent the extreme values (limits) of wear of the active area of the dental burs but cannot be used for extrapolation. There are functions that at origin and infinity have controlled behavior and, by interpolation, lead to correct interpretation for the extrapolation of the results (the thing which can be demonstrated only by experimental validation). However, as seen from the obtained results (see Table 4, Table 5 and Table 6, Figure 4), the third degree is sufficient to describe the wear phenomenon of the active part of the dental burs and establish their lifetime (durability), at least at this stage. The results closest to reality are those given by the interpolation function of the third degree (see Table 5). Furthermore, these results are in good agreement with the experimental results (see Table 6). 

Similar estimates of the durability (lifetime) of the active part of the dental burs can be made relatively, and with other parameters such as the lamellae angles, area, or radius of the peak circle, etc., of the active part of the dental burs.

For a more complete solution to the problem of the wear of dental burs, obtaining several experimental data/results, the method extension to other types of dental burs or other characteristic parameters of them, or the use of other research methods/models are necessary. In this way, the influence of this important parameter of the work regime will be highlighted/validated, *m_w_*, together with the other parameters mentioned in the paper. The mathematical model that was obtained (using the deterministic differential model for the work process of dental bur), starting from the structured, discussed, and interpolated experimental data/results of *m_w_* in this paper, will be used to investigate the possibilities of improving or optimizing the working regimes of the dental burs.

It was found that the lifetime of the dental burs studied and tested experimentally shows differences in the duration of operation depending on the drive speed, which confirms the data from the specialized literature that the cutting speed is the main determining factor of the lifetime of tools, in accordance with the principle from metal cutting technology known as “Taylor’s principle” [15].

Among the variants, which proposes them refs. [16,17,18,19] for the notion of wear and tear, we retained those related to the subject of this study: damage, degradation of an object, or the progressive change of some physical characteristics of an element of a system during operation, according to [20], whereby “wear represents the deterioration, gradual removal or deformation of material on solid surfaces”. Among the multitude of notions that define the word wear, there are also the notions of mechanical and physical erosion.

The experimental results presented in Figure 4 and Table 6, for the mass lost through wear of the studied dental burs (which represents the researched characteristic), measured by weighing showed different values obtained randomly, with each value having a number of repetitions, and so a frequency of its own.

The distribution of values (with their frequency) between the limit values of the interval can be represented by a distribution law. Usually, the range of variation of a characteristic reflects the level of manufacturing quality, in the case of a product.

As the factors that determine the dispersion of the effective dimensions (of *m_w_*) are in large numbers, of the same order, independent, and accidental, then the distribution law of the mass lost through effective wear, as a random variable (*m_w_* < *x* given and corresponds to a probability *p*), is the normal distribution law (Gaussian).

**Figure 12:**
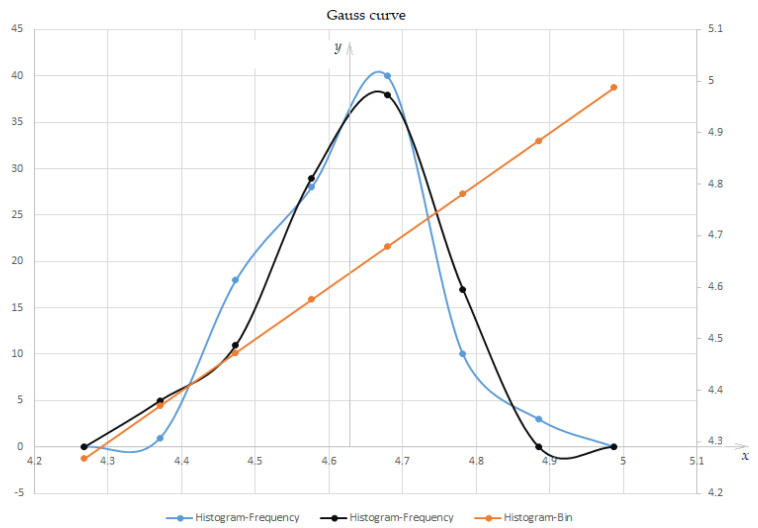
Probability density graph for the normal distribution law (Gauss).

It is known that in the series of mass production of products, in the case of dental burs, using the method of automatic processing of shape and dimensions, most of the experimental distributions approach the normal distribution, and can be considered a standard distribution. The normal distribution (Gaussian distribution), representing the distribution density, frequency function, probability distribution density, or probability density, *f*(*x*), is closest to the shape of a normal distribution function (for the blue and black color curves (see Figure 11)):

(6) $$f(x)\;=\;y=\;\frac{1}{1.22\sqrt{2\pi }}e^{-\frac{({x-11.95)}^{2}}{3}},$$

and for the red (right) curve (which represents the data set that is compared to those that led to the blue and black curves (see Figure 11)), a first-degree polynomial function:

(7) $$f(x)\;=\;y\;=x\;+\;{4e}^{-15}.$$

which has the shape of Figure 12 (bell shape, where you can also see the variation of the function *f*(*x*) = *y*) and represents the curve of the probability density function, being slightly asymmetric (to the right). This can be explained by the fact that the mass lost due to the wear of the dental burs, is measured after use (i.e., close to decommissioning) against the axis corresponding to the cluster center (around 4.63) of the deviations for the frequency distribution of the measured results (blue and black curves), but with different distribution frequencies. 

Considering the Cartesian axis system, in which the coordinate axis *y* = *f*(*x*) coincides with the straight line raised from the center of a grouping of deviations (4.63), and the peak of the Gaussian distribution curves is positioned to the right (slightly inclined to the right, see Figure 12), explains the above.

Two inflection points are observed on the probability density function curve, in the interval of abscissa 4.58 and 4.78, where the surface area under the curve represents approximately 58.33% of the total surface area (i.e., it shows where the values of the random variable are most distributed, *m_w_* < *x*. The curves tend asymptotically to the abscissa axis and show a maximum of approximately, *x* = 5.03 (blue curve) and 4.98 for *x* = 4.67, the reliability, *R*(*t*) obtained being approximately 97.42%, thus showing very good reliability. If other characteristics were also taken into account (which were not here considered), the total reliability would fall below 90%.

In the interval (4.38, 4.88), the area between the curves and the abscissa axis is approximately 97.42% of the entire area, so the intervals (−∞, 4.38) and (4.88, +∞) can be practically neglected. So, the scatter interval is 0.50 (from 4.38 to 4.88), and the limit deviations have the values (±0.25), compared to the center of the cluster, respectively; the confidence interval is (4.38, 4.88) and the critical interval is (−∞, 4.38) U (4.88, +∞).

Therefore, in this paper, by using the deterministic differential model and working methodology the wear evaluation was made possible in the work process of the active part of the analyzed dental bur, respectively, the gradual growth of lamellae wear, and rapid deterioration of the cutting edges. At the same time, the fatal failure due to increased wear rate proved the existence of a strong correlation between the rotational speed–time-wear within the different phases of the dental bur lifetime.

Thus, the gradual growth of lamellae wear, rapid deterioration of the cutting edges, and the fatal failure due to increased wear rate proved the existence of a strong correlation between the rotational speed–time-wear within the different phases of the dental bur lifetime.

Using the model allows the extension of the characteristic on other parameters of the dental bur that change in the working process as a result of wear. In addition, the methodology used is a novelty in the field, although in the literature there are various models of experimental data, considering one, two, or even three parameters in other forms and applying other mathematical methods. The model used allows the extension of the correlation/characteristic of the rotational velocity–time–wear on other parameters of the dental bur that change in the working process as a result of wear.

By the study of durability and reliability based on the wear phenomenon for the dental burs (of the same type) tested and by the experimental data, it has been shown that practical results can be obtained, such as the extension of the lifetime of the dental bur (and of the material from which the dental bur is built) or even working process optimization of the bur in operation. The experimental results/data obtained for the wear of the active part of the dental bur in the work process were validated by their interpolation and led to polynomial functions which approximate very well the dependent parameter (lost mass due to dental bur wear, *m_w_*).

The results of the interpolation established the optimal work time, with high efficiency of the dental bur, after which it can be easily decommissioned (the criterion for replacing the worn dental bur).

The researched dental burs can operate without a major risk of failure for up to 10 h, and for up to 11 h the instantaneous risk of failure is below 10%; this is very close to what was determined analytically based on the experimental results, which confirms the veracity and correlation of the analytical and experimental results.

## 5. Conclusions

- The study presented in this paper was limited to the analysis and the consideration of only one parameter, *m_w_*, to study the phenomenon of wear based on the durability and reliability of a type of dental bur.
- The wear phenomenon limits the durability and reliability of the active part of a dental bur, by the parameter, *m_w_*, considered in the experimental program, studied based on the experimental results obtained in the work process and validated by polynomial interpolation
- Polynomial interpolation of the experimental data/results allowed the determination of the limit (extreme) wear values, when a dental bur becomes ineffective (i.e., must be replaced) and led to practical applications, useful for the determining of its lifetime, even for the optimization of the parameters, *m_w_*.
- Additionally, the results obtained by interpolation showed that the dental bur during the work process has a high efficiency, and the limit of use of a dental bur was establish by the determination durability and reliability, to ensure high dental-material-processing standards.
- The results closest to reality are those given by the interpolation function of the third degree, and they are in good agreement with the experimental results, but the complete solution of this problem without experimental information becomes an approximate one because the experiments were performed on only one type of dental bur.
- The researched parameter *m_w_*, measured by weighing, was observed to take different values at random between two limits; each value has its own frequency (a number of repetitions), and the distribution of these values (with their frequency) can be represented by a normal distribution law based on Gauss’s function.
- Similar studies can be justified and use other functional parameters of the active part of the researched dental bur to check the validity of the method used, regarding the lifetime (durability) and optimization of their operation (reliability).
- The continuation of the research method, by obtaining several experimental data and additional information for a more complete solution to the problem of wear of the active part of dental burs, method extension on other functional parameters that were not monitored, and other types of dental burs in order to record the parameters, is proposed, using new deterministic or even statistical models of the wear phenomenon.
- In this way, the influence (importance) of the parameter, *m_w_*, along with other characteristic/functional parameters mentioned in the paper will be compared and validated, and experimental data structured and discussed will be modeled and used to investigate the possibilities of improving or optimizing the working regimes of dental burs.

## Figures and Tables

**Table 1:** Work process parameters.

Parameter	Notation	Unit of Measurement	Parameter Type
Time	*t*	s, min, h	Command
Mass lost by dental bur wear	*m_w_*	g	Input/Output
Milled mass (of dental material)	*m_m_*	g	Qualitative
Advance (Feed)	*s*	mm	Command
Rotational speed of dental bur	*n/ω*	rpm/s^−1^	Command

**Table 2:** Mass and the dimensions of the specimen dental bur researched.

Type of Bur/Features	Mass, *m_f_* [g]	Total Length, [mm]	Length of the Active Part, [mm]	Average Diameter, [mm]	Maximum Diameter, [mm]
Specimen dental bur	4.701	52.750	14.500	5.400	6.200

**Table 3:** Mass and the dimensions of the reference/sample dental material subjected to processing.

Name	Mass, *m_m_* [g]	Total Length, [mm]	Diameter, [mm]
Sample dental material	6.18	13.00	8.60

**Table 4:** Coefficients and interpolation errors for parameters *t* and ω.

	*m_w_*—Values by Interpolation of the Degree Polynomial (Function):		
Polynomial Coefficients	Degree I	Degree II	Degree III
*c*_00_	−0.315	−0.003346	0
*c*_10_	1.746·10^−5^	−6.150·10^−5^	6.744·10^−5^
*c*_01_	3.866·10^−4^	2.275·10^−4^	−5.699·10^−4^
*c*_11_	0	1.333·10^−8^	−1.028·10^−8^
*c*_20_	0	3.792·10^−9^	−8.736·10^−9^
*c*_02_	0	1.518·10^−8^	6.156·10^−7^
*c*_21_	0	0	1.762·10^−12^
*c*_12_	0	0	−1.82·10^−12^
*c*_30_	0	0	3.37·10^−13^
*c*_03_	0	0	−1.038·10^−10^
*ε* %	7.828	1.733	0.486

*Note:* Polynomial coefficients were calculated with Mathcad using the least squares method.

**Table 5:** A lifetime of the active part of the dental bur, estimated for *m_w_* at four working speeds and three interpolation functions.

Rotation Speed, [rpm]	Lifetime (Durability) in Hours, for *m_w_*, Given by the Polynomial Interpolation Function of:		
	Degree I	Degree II	Degree III
7000	12.753	11.720	11.517
12,000	8.558	7.886	7.824
20,000	5.998	5.984	5.795
35,000	3.623	3.597	3464

**Table 6:** A calculated lifetime of the active part of dental bur used in experimental research.

No. Crt.	Rotational Speed of Dental Bur, Rpm	Lifetime (Duration). Calculated for *m_w_*, Hours
1	7000	11.373
2		10.265
3		12.613
4		11.214
5		11.620
6	12,000	7.634
7		7.488
8		8.358
9		7.354
10		7.786
11	20,000	5.898
12		5.911
13		5.098
14		5.684
15		5.884
16	35,000	3.275
17		3.153
18		3.523
19		3.497
20		3.372
Average time values of calculated life, hours	7000	11.417
	12,000	7.724
	20,000	5.695
	35,000	3.364

## Data Availability

Data are contained within the article.
